# *In vitro* evaluation of *Lactiplantibacillus plantarum* HOKKAIDO strain, effective lactic acid bacteria for calf diarrhea

**DOI:** 10.3389/fvets.2023.1145445

**Published:** 2023-04-05

**Authors:** Mari Ikehata, Satoru Konnai, Tomohiro Okagawa, Kentaro Abe, Mitsuru Honma, Toru Kitamura, Naoya Maekawa, Yasuhiko Suzuki, Shiro Murata, Kazuhiko Ohashi

**Affiliations:** ^1^Department of Disease Control, Faculty of Veterinary Medicine, Hokkaido University, Sapporo, Japan; ^2^Department of Advanced Pharmaceutics, Faculty of Veterinary Medicine, Hokkaido University, Sapporo, Japan; ^3^Institute for Vaccine Research and Development (HU-IVReD), Hokkaido University, Sapporo, Japan; ^4^Hokkaido Research Station, Snow Brand Seed Co., Ltd., Naganuma, Japan; ^5^Division of Bioresources, International Institute for Zoonosis Control, Hokkaido University, Sapporo, Japan; ^6^Global Station for Zoonosis Control, Global Institution for Collaborative Research and Education (GI-CoRE), Hokkaido University, Sapporo, Japan; ^7^International Affairs Office, Faculty of Veterinary Medicine, Hokkaido University, Sapporo, Japan

**Keywords:** *Lactiplantibacillus plantarum* HOKKAIDO strain, monocytes–cell, TLR2/4, antiviral effects, rotavirus

## Abstract

Calf diarrhea adversely affects growth and sometimes results in mortality, leading to severe economic losses to the cattle industry. Antibiotics are useful in the treatment against bacterial diarrhea, but not against viral, protozoan, and antibiotic-resistant bacterial diarrhea. Therefore, there are growing requirements for a novel control method for calf diarrhea. Probiotics have been considered promising candidates for preventive and supportive therapy for calf diarrhea for many years. A recent study has revealed that *Lactiplantibacillus plantarum* HOKKAIDO strain (Lp-HKD) reduces intestinal pathology and the severity of diarrhea in bovine rotavirus (BRV)-infected calves. Lp-HKD is known to enhance the function of human immune cells and expected to be used as probiotics for humans. Therefore, it is hypothesized that Lp-HKD modulates antiviral immune response in cattle and provide the clinical benefits in BRV-infected calves. However, the detailed mechanism of Lp-HKD-induced immunomodulation remains unknown. Thus, this study aimed to elucidate the immunomodulatory and antiviral effects of Lp-HKD in cattle. Cultivation assay of bovine peripheral blood mononuclear cells (PBMCs) showed that live and heat-killed Lp-HKD stimulates the production of interleukin-1β (IL-1β), IL-6, IL-10, and interferon-γ (IFN-γ) from PBMCs. Stimulation by heat-killed Lp-HKD yielded stronger cytokine production than stimulation by the live Lp-HKD. Additionally, CD14^+^ monocytes were identified as major producers of IL-1β, IL-6, and IL-10 under Lp-HKD stimulation; however, IFN-γ was mainly produced from immune cells other than CD14^+^ monocytes. Depletion of CD14^+^ monocytes from the PBMCs cultivation strongly decreased cytokine production induced by heat-killed Lp-HKD. The inhibition of toll-like receptor (TLR) 2/4 signaling decreased IL-1β and IL-6 production induced by live Lp-HKD and IL-1β, IL-6, and IFN-γ production induced by heat-killed Lp-HKD. Furthermore, live or heat-killed Lp-HKD also activated T cells and their production of IFN-γ and tumor necrosis factor-α. Then, culture supernatants of bovine PBMCs treated with heat-killed Lp-HKD demonstrated antiviral effects against BRV *in vitro*. In conclusion, this study demonstrated that Lp-HKD activates the functions of bovine immune cells *via* TLR2/4 signaling and exerts an antiviral effect against BRV through the induction of antiviral cytokines. Lp-HKD could be useful for the prevention and treatment of calf diarrhea through its immune activating effect.

## Introduction

Lactic acid bacteria (LAB) such as *Lactobacillus* spp., *Lactiplantibacillus* spp., *Lactococcus* spp., and *Streptococcus* spp. have been studied as probiotic bacteria (1, 2). A previous report indicated the use of LAB to prevent infectious diseases in newborn babies in rural India, where many children are still dying from these diseases (3). *Lactiplantibacillus plantarum* HOKKAIDO strain (Lp-HKD) is a probiotic LAB isolated from well-pickled vegetables in Hokkaido, Japan (4). Lp-HKD induces the production of interleukin (IL)-6, IL-10, IL-12, and tumor necrosis factor-α (TNF-α) by human dendritic cells (5). Furthermore, a previous study reported that heat-killed Lp-HKD alleviated clinical symptoms of the common cold in humans (6). Therefore, Lp-HKD is expected to improve antiviral immune function in humans.

Diarrhea is a common disease observed in calves, which is caused by viral, bacterial, and protozoan infections, as well as non-infectious factors such as dietary and nervous factors. Among these factors, bovine rotavirus (BRV) infection and bovine cryptosporidiosis, which are prevalent among cattle in Japan, cause severe diarrhea in calves (7). BRV infects cattle of all ages; however, a higher incidence of enteritis, more severe clinical signs, and higher mortality are observed in calves (8). When calves develop diarrhea, growth retardation occurs even if they survive, leading to severe economic losses (9). Although antibiotics are useful to the treatment of bacterial diarrhea (10), they have no direct effect on viral, protozoan, and antibiotic-resistant bacterial diarrhea (11). Therefore, the development of a novel alternative preventive strategy for bovine diarrhea is required, and the use of probiotics has been considered as a promising candidate. Indeed, feeding probiotics was reported to reduce the incidence of diarrhea and have beneficial effects on calf growth (12). Furthermore, a previous study reported that feeding milk replacer (MR) supplemented with highly-concentrated Lp-HKD reduced diarrhea induced by BRV challenge and tissue damage to the intestinal tract in newborn calves (13). Although the mechanism of the preventive effects of Lp-HKD remains unknown, we hypothesized that Lp-HKD modulated bovine immune responses to viral infections and reduced the severity of diarrhea.

In this study, we investigated the immunostimulatory and antiviral effects of Lp-HKD to elucidate the mechanisms of immunomodulation by Lp-HKD in cattle. We examined the immunomodulatory mechanisms involving not only live Lp-HKD but also heat-killed Lp-HKD.

## Materials and methods

### Blood samples

Peripheral blood samples of cattle were obtained from adult female Holstein-breed cattle in dairy farms in Hokkaido, Japan. The animal experiments in this study were approved by the Institutional Animal Care and Use Committee of Hokkaido University (approval numbers: 17-0024 and 18-0147) and performed in accordance with the relevant guidelines and regulations of the Faculty of Veterinary Medicine of Hokkaido University, which is fully accredited by the Association for Assessment and Accreditation of Laboratory Animal Care International. Written informed consent was obtained from the owners for the participation of their animals in this study.

### Preparation of Lp-HKD

Lp-HKD (Food Processing Research Center, Hokkaido Research Organization, Ebetsu, Japan; Japanese patent No. 3925502) was cultured in MRS broth (BD Biosciences, San Jose, CA, USA) at 37°C for 24 h in a 1 L bottle and collected by centrifugation at 5,800 × *g* for 15 min at 10°C. The bacteria were washed twice with phosphate-buffered saline (PBS, pH 7.2) and finally resuspended in PBS. The bacteria were plated on MRS agar plate (BD Biosciences) and anaerobically incubated at 37°C for 17–24 h and colony-forming unit (CFU) were counted. The live Lp-HKD was stored at 4°C and used for further experiments within 7 days. Heat-killed Lp-HKD was prepared by heating the bacteria at 96°C for 10 min and stored at −30°C until further experiments. Successful heat-killing was confirmed by the absence of bacterial growth on the MRS agar plate (BD Biosciences).

### Cell preparation

Bovine peripheral blood mononuclear cells (PBMCs) were purified from blood samples by density gradient centrifugation using Percoll (GE Healthcare, Little Chalfont, UK). CD14^+^ cells were freshly isolated from PBMCs using the autoMACS Pro System (Miltenyi Biotec, Bergisch Gladbach, Germany) with anti-bovine CD14 mAb (CAM36A, Washington State University Monoclonal Antibody Center, Pullman, WA, USA), and anti-mouse IgG_1_ MicroBeads (Miltenyi Biotec) as described previously with some modification (14). CD14^−^ PBMCs were prepared from negative fractions of CD14^+^ cell sorting. The purity of each cell fraction was confirmed using FACS Verse (BD Biosciences) or FACS Lyric (BD Biosciences). Highly pure populations (>95%) were used for experiments.

### Cell cultivation assay

PBMCs (4 × 10^6^ cells/mL), CD14^+^ cells (2 × 10^6^ cells/mL), or CD14^−^ PBMCs (4 × 10^6^ cells/mL) were seeded into 96-well round-bottom plate (Corning Inc., Corning, NY, USA) with the RPMI 1640 medium (Sigma-Aldrich) containing 10% heat-inactivated FBS (Thermo Fisher Scientific, Waltham, MA, USA), 200 IU/mL penicillin (Thermo Fisher Scientific), 200 μg/mL streptomycin (Thermo Fisher Scientific), and 0.01% L-glutamine (Thermo Fisher Scientific). Then, the cells were cultured in the presence of live Lp-HKD (PBMCs: 4 × 10^6^ CFU/mL, CD14^+^ cells: 2 × 10^6^ CFU/mL) or heat-killed Lp-HKD (PBMCs and CD14^−^ PBMCs: 4 × 10^6^ CFU/mL, CD14^+^ cells: 2 × 10^6^ CFU/mL) at 37°C under 5% CO_2_ for 17 h.

To investigate the effects of blocking toll-like receptors (TLR) 2/4 signaling, PBMCs (4 × 10^6^ cells/mL) were incubated with 10 μM sparstolonin B (SsnB; Sigma-Aldrich), as described previously with a slight modification in the concentration of SsnB (15, 16), in the presence of live or heat-killed Lp-HKD (4 × 10^6^ CFU/mL) in a 96-well plate (Corning Inc.) at 37°C under 5% CO_2_ for 17 h. In this study, the optimal concentration of SsnB for the stimulation of bovine PBMCs was determined as 10 μM. Dimethyl sulfoxide (DMSO; Nacali Tesque, Kyoto, Japan) was used as a vehicle control of SsnB.

### Quantification of cytokines by ELISA

To investigate whether the Lp-HKD stimulations and TLR2/4 signaling promote cytokine production, culture supernatants of PBMCs, CD14^+^ cells, or CD14^−^ PBMCs were collected, and the concentrations of IL-1β, IL-6, and interferon (IFN)-γ were measured using the Bovine IL-1β ELISA Reagent Kit (Thermo Fisher Scientific), the Bovine IL-6 ELISA Reagent Kit (Thermo Fisher Scientific), and the Bovine IFN-γ ELISA Development Kit (Mabtech, Nacka Strand, Sweden), respectively, according to the manufacturers' protocols. The concentration of IL-10 was measured as described previously (17). Briefly, sandwich ELISA of IL-10 was performed using anti-bovine IL-10 (CC318; Bio-Rad, Hercules, CA, USA) as a capture antibody and biotinylated anti-bovine IL-10 (CC320; Bio-Rad) as a detection antibody.

### Flow cytometric analysis of T cells

To investigate whether the Lp-HKD stimulations activate bovine T cells, PBMCs (4 × 10^6^ cells/mL) were incubated with live or heat-killed Lp-HKD as described above. To examine the effects of Lp-HKD on T-cell activation in the cultivated PBMCs, the expression of CD25 and CD69 were analyzed by flow cytometry. The cultured PBMCs were harvested and blocked with PBS containing 10% goat serum (Thermo Fisher Scientific) at 25°C for 15 min. After washing, the cells were stained with PerCp/Cy5.5-conjugated anti-CD3 mAb (MM1A; Washington State University Monoclonal Antibody Center), PE/Cy7-conjugated anti-CD4 mAb (CC8; Bio-Rad), PE-conjugated anti-CD8 mAb (CC63; Bio-Rad), Alexa Fluor 488-labeled anti-CD25 mAb (IL-A111; Bio-Rad), Alexa Fluor 647-labeled anti-CD69 mAb (KTSN7A; Washington State University Monoclonal Antibody Center), and Fixable Viability Dye eFluor 780 (Thermo Fisher Scientific) at 4°C for 20 min. MM1A and CC8 were conjugated with PerCp/Cy5.5 and PE/Cy7, respectively, using the Lightning-Link Conjugation Kits (Abcam, Cambridge, UK). IL-A111 and KTSN7A were prelabeled using the Zenon Alexa Fluor 488 and Zenon Alexa Fluor 647 Mouse IgG_1_ Labeling Kits (Thermo Fisher Scientific), respectively. The stained cells were washed and analyzed immediately using FACS Verse (BD Biosciences). Antibody dilution and cell washing were performed with PBS containing 1% bovine serum albumin (Sigma-Aldrich) (BSA-PBS).

To examine the effect of Lp-HKD on cytokine production in T cells, PBMCs were cultured for 17 h, and 10 μg/mL of Brefeldin A (Sigma-Aldrich) was added for last 5 h. The cells were collected and the blocking was performed as described above. Then, the cells were stained with PerCp/Cy5.5-conjugated anti-CD3 mAb (MM1A), Alexa Flour 647-conjugated anti-CD4 mAb (CC8; Bio-Rad), FITC-conjugated anti-CD8 mAb (CC63; Bio-Rad), and Fixable Viability Dye eFluor 780 (Thermo Fisher Scientific) in 1% BSA-PBS at 4°C for 20 min. After washing, the cells were fixed using Fixation Buffer (BioLegend, San Diego, CA, USA) at 4°C for 20 min and permeabilized using Perm/Wash Buffer (BioLegend) at 4°C for 15 min. After washing, the cells were stained with PE-conjugated anti-IFN-γ mAb (CC302; Bio-Rad) and biotinylated anti-bovine TNF-α mAb (CC328; Bio-Rad) in Perm/Wash Buffer (BioLegend) at 4°C for 20 min. Then, the cells were washed and labeled with PE/Cy7-conjugated streptavidin (Thermo Fisher Scientific) at 4°C for 20 min. The stained cells were washed and analyzed immediately using FACS Lyric (BD Biosciences).

### Viral titer assay

To assess the antiviral effect of soluble factors produced from PBMCs, PBMCs were incubated with or without heat-killed Lp-HKD for 17 h as described above, and culture supernatants were collected and filtrated through a 0.22-μm syringe filter (Merck Millipore, Burlington, MA, USA) to remove bacteria and cells.

BRV (Lincoln strain) was activated at 37°C under 5% CO_2_ for 1 h in E-MEM (FUJIFILM Wako Pure Chemical, Osaka, Japan) containing 0.02% acetylated trypsin (Sigma-Aldrish). MA104 cells, which are a monkey kidney epithelial cell line and are highly susceptible to rotavirus including BRV (18), were seeded in 24-well plates (Corning Inc.) and infected with 200 μL of the activated BRV, which contains 4.31 copies of the virus, with shaking at 37°C under 5% CO_2_ for 1 h. After absorption, 300 μL of E-MEM (FUJIFILM Wako Pure Chemical) containing 200 IU/mL penicillin (Thermo Fisher Scientific), 200 μg/mL streptomycin (Thermo Fisher Scientific), and 0.01% L-glutamine (Thermo Fisher Scientific), and 300 μL of the culture supernatants of PBMCs collected as described above were added on the infected cell monolayers and the cells were cultured at 37°C under 5% CO_2_ for five days. The cell-free culture supernatants were collected every day. Four independent culture wells were incubated for each sample.

To measure BRV titer, viral RNA (vRNA) was extracted from the culture supernatants using the QIAamp Viral RNA Mini Kit (Qiagen, Hilden, Germany) according to the manufacturer's instructions. The quantitative reverse transcription polymerase chain reaction (qRT-PCR) assay was performed as previously described, with slight modifications (19). NSP5 gene of rotavirus A in each RNA sample was quantified in duplicate using SuperScript III Platinum One-Step qRT-PCR Kit (Thermo Fisher Scientific), gene specific primers (5'-TTCTGCTTCAAACGAYCCACTC-3' and 5'-GAGAAATCYACTTGRTCGCA-3'), and a probe (5'-FAM-TCCATAGAYACRCCAGYRTCTGCRTTTGTC-BHQ-3') with a LightCycler 480 System II (Roche Diagnostics, Mannheim, Germany). The PCR condition was 50°C for 40 min (for RT), followed by the amplification of the template by PCR for 45 cycles at 95°C for 15 s and 60°C for 60 s. 10^8^-10^2^ copies of *NSP5* RNA of Lincoln strain were used to generate calibration curves in duplicate and reported values are the average numbers of viral copies per 1 mL of culture supernatant.

### Statistical analysis

Differences were determined using the Wilcoxon signed-rank test and Welch's *t*-test for two-group comparisons, and the Steel-Dwass test for multiple-group comparisons using JMP Pro 16.2.0 (SAS Institute, Cary, NC, USA). A *p* < 0.05 was considered statistically significant.

## Results

### Activation of immune responses by Lp-HKD

**Figure 1:**
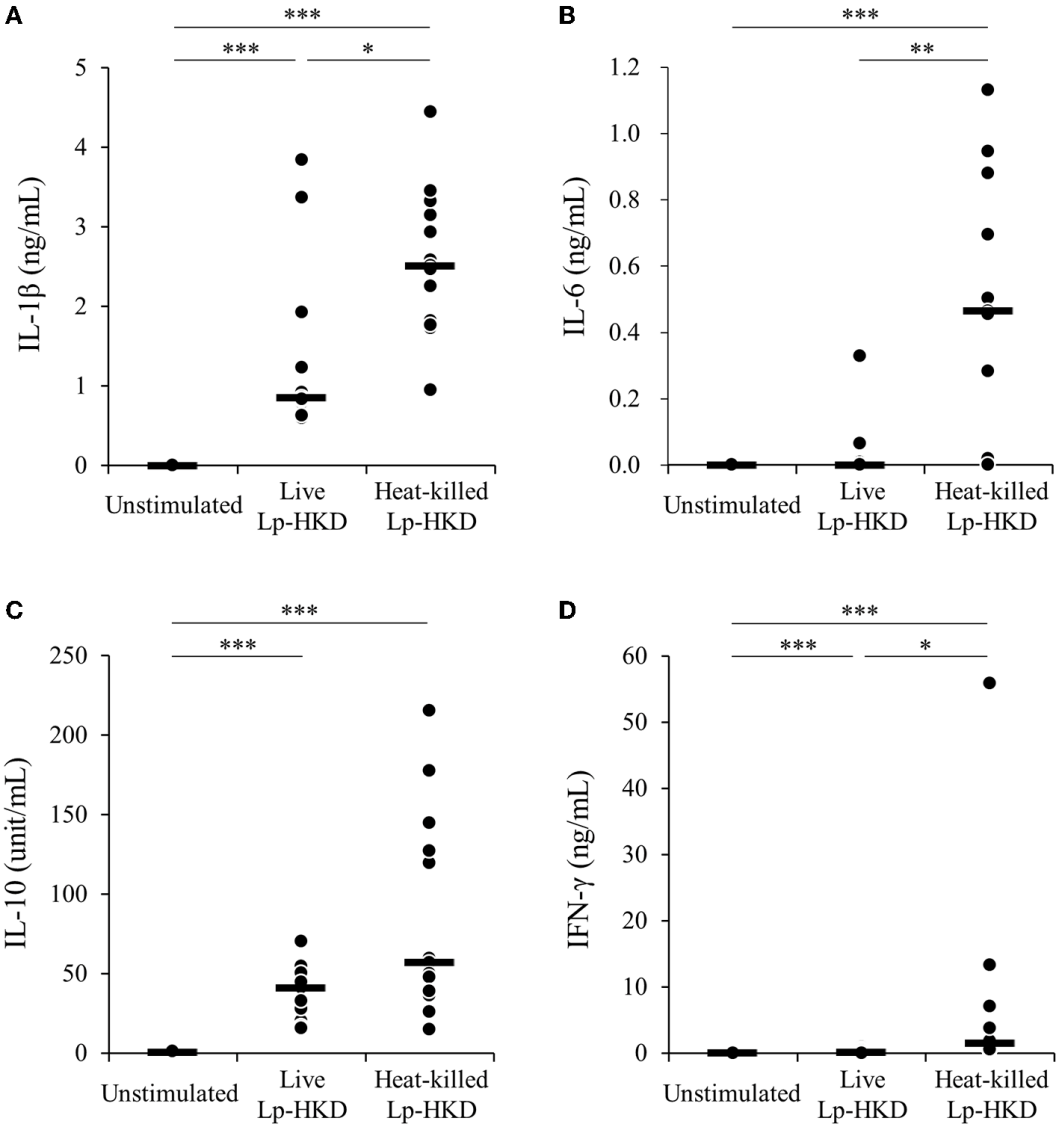
Lp-HKD-induced cytokine production by PBMCs. **(A–D)** Bovine PBMCs were incubated with either live or heat-killed Lp-HKD (4 × 10^6^ CFU/mL) or without stimulation for 17 h. The concentrations of IL-1β [**(A)**, *n* = 13], IL-6 [**(B)**, *n* = 13], IL-10 [**(C)**, *n* = 13], and IFN-γ [**(D)**, *n* = 13] in culture supernatants were determined by ELISA. Statistical significance was determined by the Steel-Dwass test. *p < 0.05, **p < 0.01, ***p < 0.001.

To examine whether Lp-HKD activates immune responses in cattle, bovine PBMCs were cultured either with live or heat-killed Lp-HKD or without stimulation, and the levels of IL-1β, IL-6, IL-10, and IFN-γ in culture supernatants were measured by ELISA. Stimulation with live Lp-HKD increased the production of IL-1β, IL-10, and IFN-γ (Figures 1A, C, D). Stimulation with heat-killed Lp-HKD increased the production of IL-1β, IL-6, IL-10, and IFN-γ (Figures 1A–D). Additionally, heat-killed Lp-HKD significantly induced the production of IL-1β, IL-6, and IFN-γ compared with live Lp-HKD (Figures 1A, B, D). These results suggest that Lp-HKD, especially heat-killed bacteria, activates cytokine production in bovine immune cells.

### Induction of immune responses by Lp-HKD in CD14^+^ monocytes

**Figure 2:**
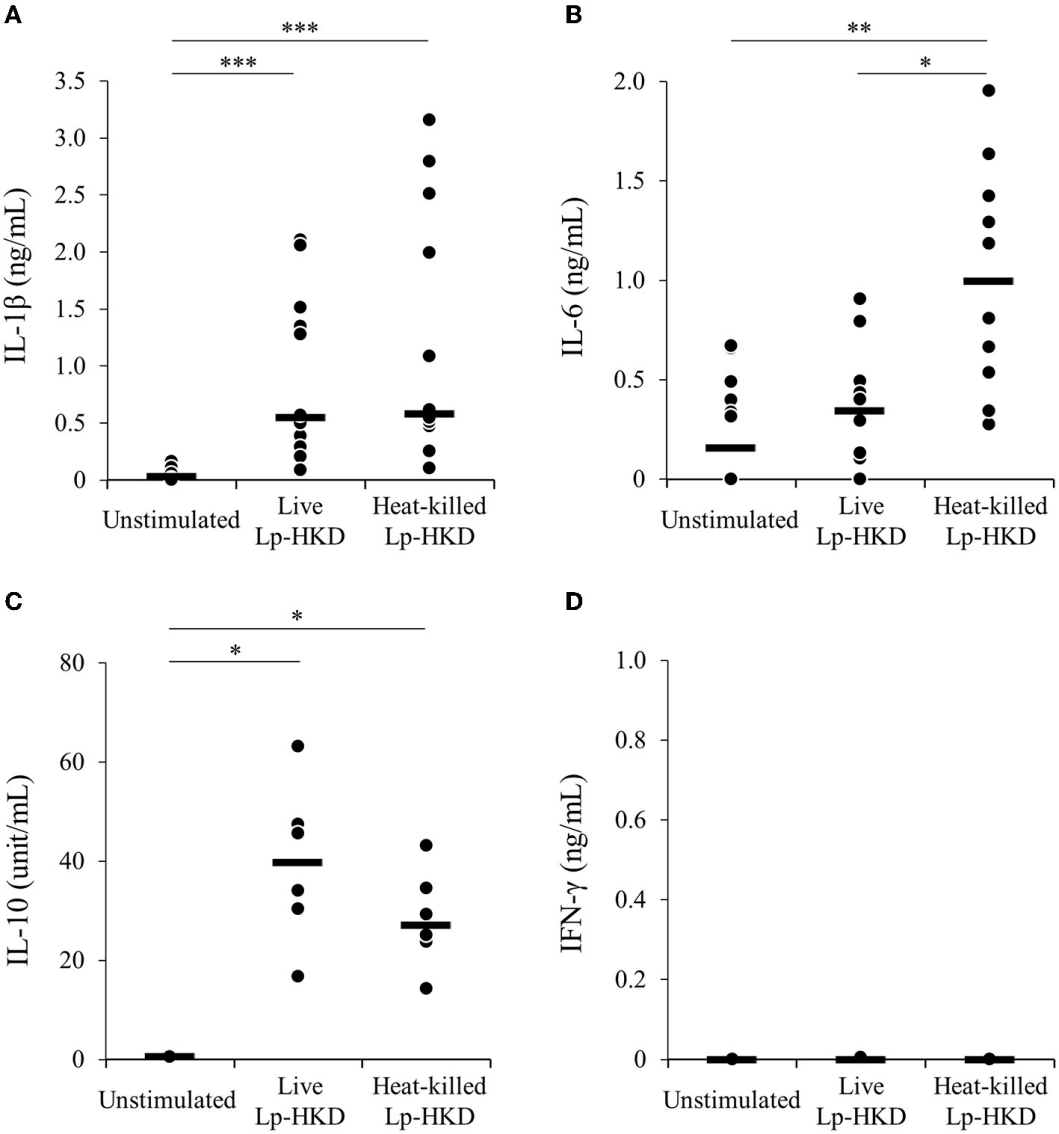
Lp-HKD-induced cytokine production by CD14^+^ monocytes. **(A–D)** CD14^+^ monocytes were incubated with either live or heat-killed Lp-HKD (2 × 10^6^ CFU/mL) or without stimulation for 17 h. The concentrations of IL-1β [**(A)**, *n* = 12], IL-6 [**(B)**, *n* = 12], IL-10 [**(C)**, *n* = 6], and IFN-γ [**(D)**, *n* = 6] in culture supernatants were determined by ELISA. Statistical significance was determined by the Steel-Dwass test. *p < 0.05, **p < 0.01, ***p < 0.001.

To examine whether Lp-HKD stimulates CD14^+^ monocytes, CD14^+^ monocytes were cultured either with live or heat-killed Lp-HKD or without stimulation, and the cytokine productions in culture supernatants were measured by ELISA. Stimulation with live Lp-HKD increased the production of IL-1β and IL-10 (Figures 2A, C). Stimulation with heat-killed Lp-HKD increased the production of IL-1β, IL-6, and IL-10 (Figures 2A–C). Additionally, heat-killed Lp-HKD significantly induced the production of IL-6 compared with live Lp-HKD (Figure 2B). In contrast, the production of IFN-γ was below the detection limit in all groups, and there was no significant difference between groups (Figure 2D).

**Figure 3:**
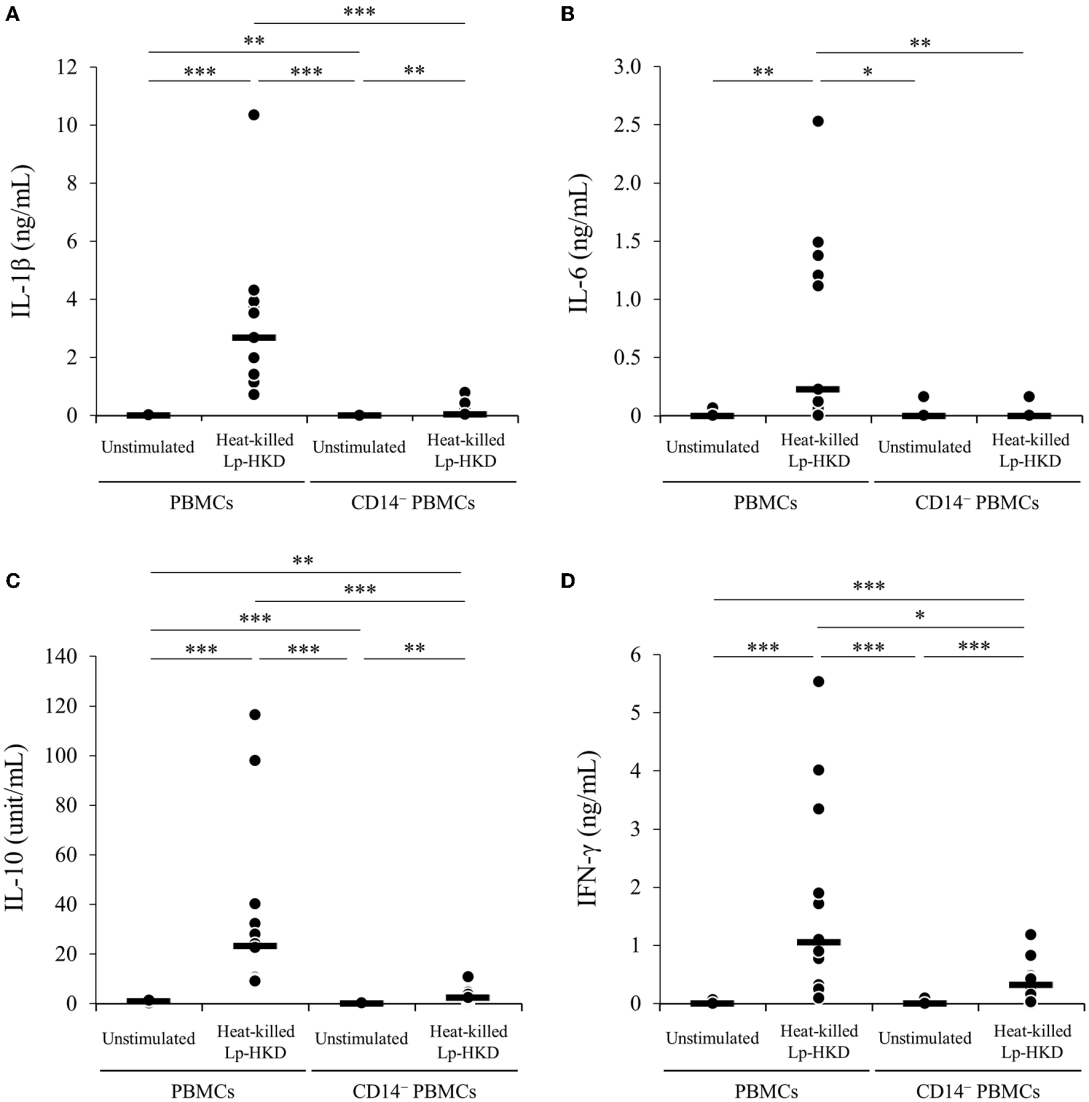
Lp-HKD-induced cytokine production by CD14^−^ PBMCs. **(A–D)** CD14^−^ PBMCs were incubated with or without heat-killed Lp-HKD (4 × 10^6^ CFU/mL) for 17 h. The concentrations of IL-1β [**(A)**, *n* = 12], IL-6 [**(B)**, *n* = 12], IL-10 [**(C)**, *n* = 12], and IFN-γ [**(D)**, *n* = 12] in culture supernatants were determined by ELISA. Statistical significance was determined by the Steel-Dwass test. *p < 0.05,**p < 0.01,***p < 0.001.

Furthermore, to examine whether CD14^+^ monocyte is responsible for the activation of immune responses against Lp-HKD in bovine PBMCs, CD14^−^ PBMCs were cultured with or without heat-killed Lp-HKD, and the cytokine productions in culture supernatants were measured by ELISA. The production of IL-1β, IL-6, IL-10, and IFN-γ by CD14^−^ PBMCs was significantly decreased when compared with those from PBMCs containing CD14^+^ monocytes (Figures 3A–D). Collectively, these results indicate that CD14^+^ monocytes could be a major cell type producing IL-1β, IL-6, and IL-10 against Lp-HKD. On the other hand, IFN-γ is produced by immune cells other than CD14^+^ monocytes although CD14^+^ monocytes are required to produce IFN-γ by these immune cells in response to Lp-HKD.

### Induction of immune responses by TLR2/4

**Figure 4:**
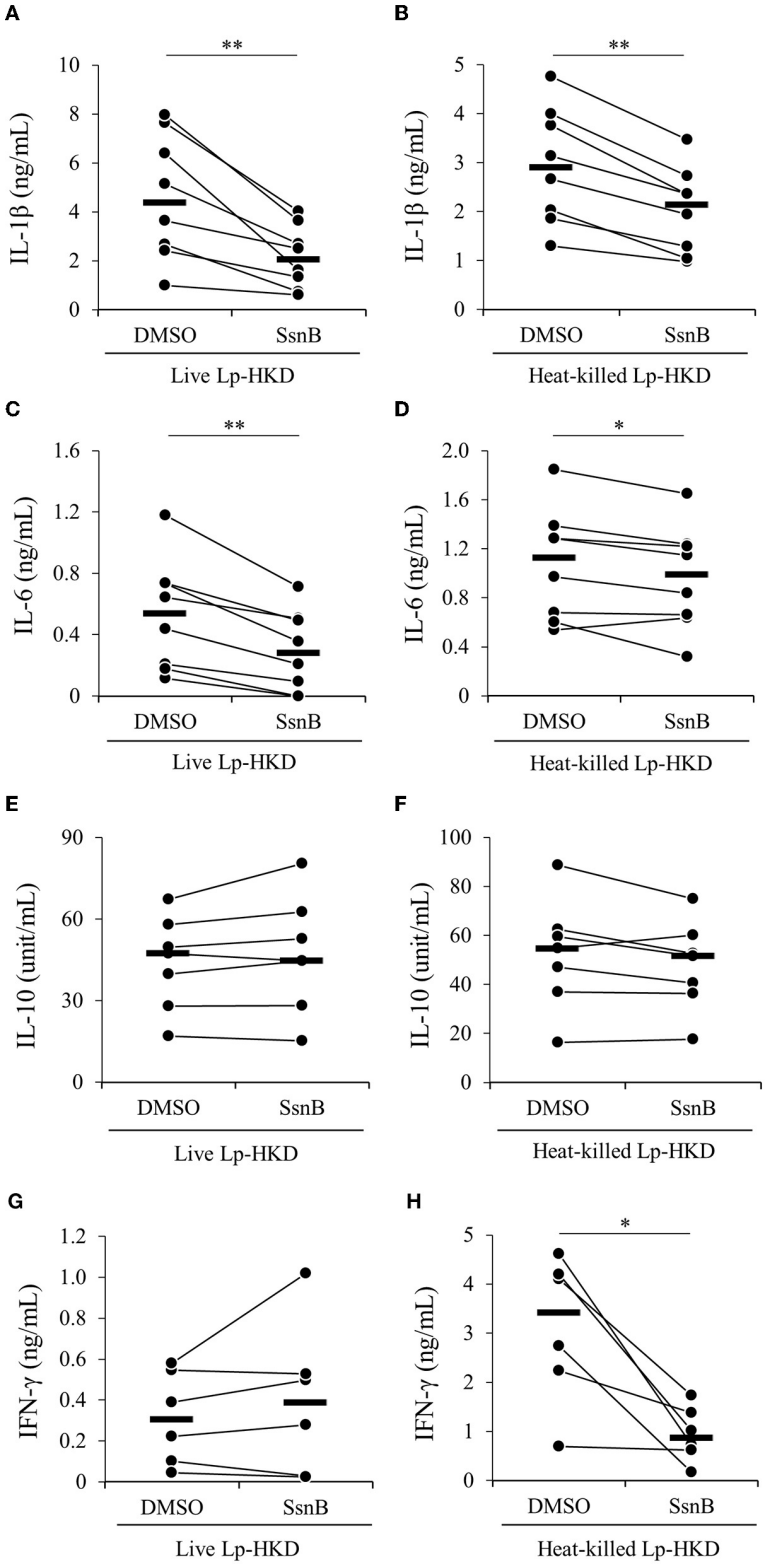
Lp-HKD-induced cytokine production by PBMCs treated with the TLR2/4 antagonist. PBMCs were incubated with live Lp-HKD (4 × 10^6^ CFU/mL) **(A, C, E, G)** or heat-killed Lp-HKD (4 × 10^6^ CFU/mL) **(B, D, F, H)** or without stimulation under the inhibition of TLR2/4 signaling by SsnB. The concentrations of IL-1β [**(A, B)**, *n* = 8], IL-6 [**(C, D)**, *n* = 8], IL-10 [**(E, F)**, *n* = 7], and IFN-γ [**(G, H)**, *n* = 6] in culture supernatants were determined by ELISA. Statistical significance was determined by the Wilcoxon signed-rank test. *p < 0.05, **p < 0.01.

To determine whether Lp-HKD stimulates TLR2/4 signaling, PBMCs were incubated with a selective antagonist of TLR2/4 (SsnB) in the presence of live or heat-killed Lp-HKD, and the cytokine productions in culture supernatants were measured by ELISA. The inhibition of TLR2/4 signaling decreased the production of IL-1β and IL-6 induced by live Lp-HKD (Figures 4A, C). However, no significant difference in the production of IL-10 and IFN-γ was observed in the TLR2/4 inhibition in PBMCs stimulated with live Lp-HKD (Figures 4E, G). In PBMCs stimulated with heat-killed Lp-HKD, the inhibition of TLR2/4 signaling decreased the production of IL-1β, IL-6, and IFN-γ (Figures 4B, D, H). On the other hand, no significant difference was observed in the production of IL-10 by the TLR2/4 inhibition (Figure 4F). These results suggest that Lp-HKD induces cytokine production mainly *via* the TLR2/4 signaling on bovine immune cells.

### T-cell activation by Lp-HKD

**Figure 5:**
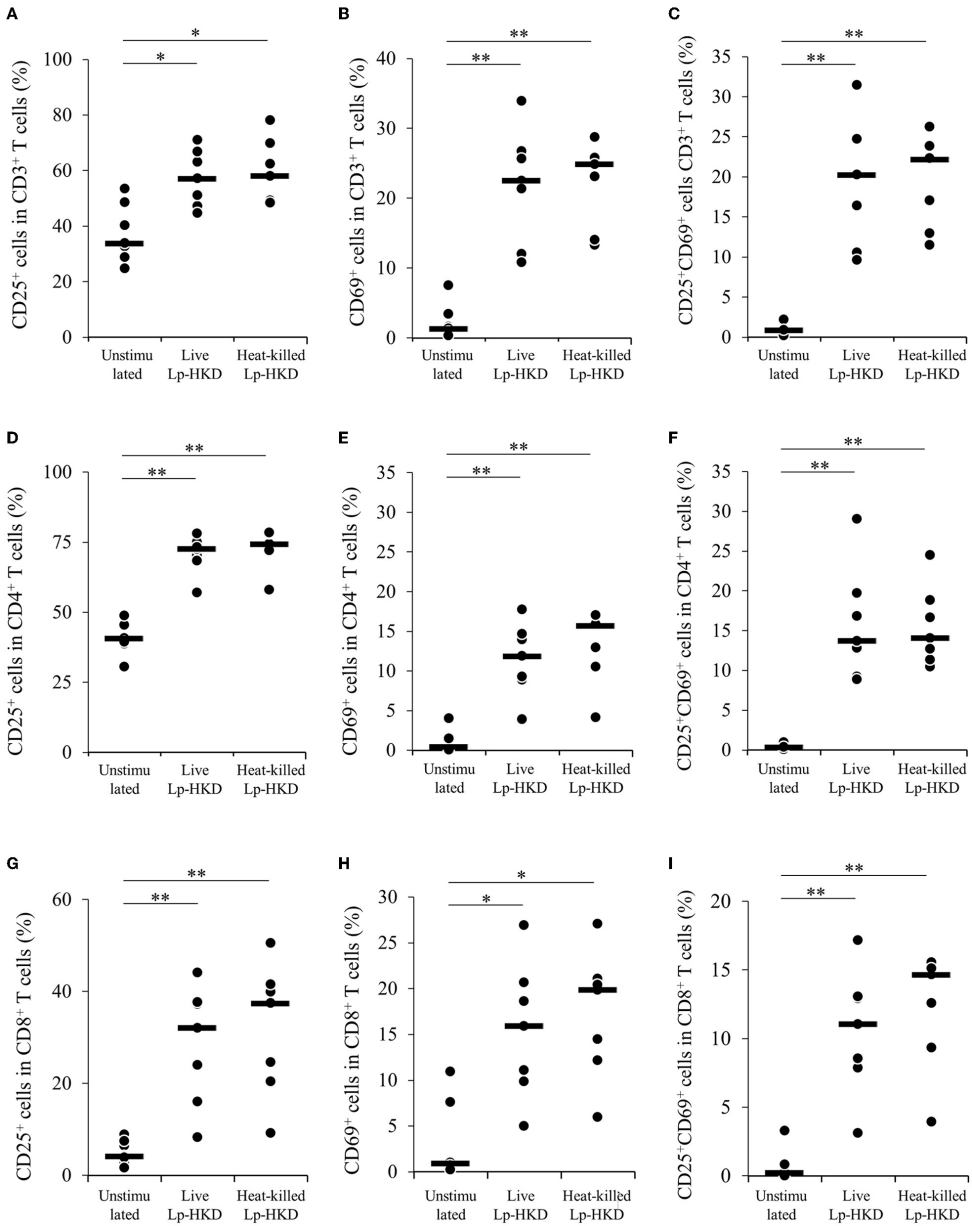
T-cell activation by Lp-HKD. **(A–I)** PBMCs were incubated with live or heat-killed Lp-HKD (4 × 10^6^ CFU/mL) or without stimulation for 17 h. The expression of CD25 and CD69 in CD3^+^ T cells [**(A–C)**, *n* = 7], CD3^+^CD4^+^ cells [**(D–F)**, *n* = 7] and CD3^+^CD8^+^ T cells [**(G–I)**, *n* = 7] was assayed by flow cytometry. Statistical significance was determined by the Steel-Dwass test. *p < 0.05, **p < 0.01.

Because Lp-HKD induced IFN-γ production from immune cells other than CD14^+^ monocytes (Figures 2B, D), we hypothesized that Lp-HKD also activated T-cell responses. We examined the effects of Lp-HKD on the activation of T-cell responses by the PBMC cultivation assays. PBMCs were cultured either with live or heat-killed Lp-HKD, and the expression of activation markers CD25 and CD69 was analyzed on T cells by flow cytometry. As shown in Supplementary Figure S1, CD3^+^, CD3^+^CD4^+^, CD3^+^CD8^+^ T cells were gated in live lymphocytes and then analyzed for expression of CD25 and CD69. The percentages of CD25^+^, CD69^+^, and CD25^+^CD69^+^ cells in CD3^+^ T cells were higher in PBMCs cultured with live or heat-killed Lp-HKD than without Lp-HKD (Figures 5A–C). Furthermore, stimulation with live and heat-killed Lp-HKD increased the percentages of CD25^+^, CD69^+^, and CD25^+^CD69^+^ cells in both CD3^+^CD4^+^ and CD3^+^CD8^+^ T cells (Figures 5D–I).

**Figure 6:**
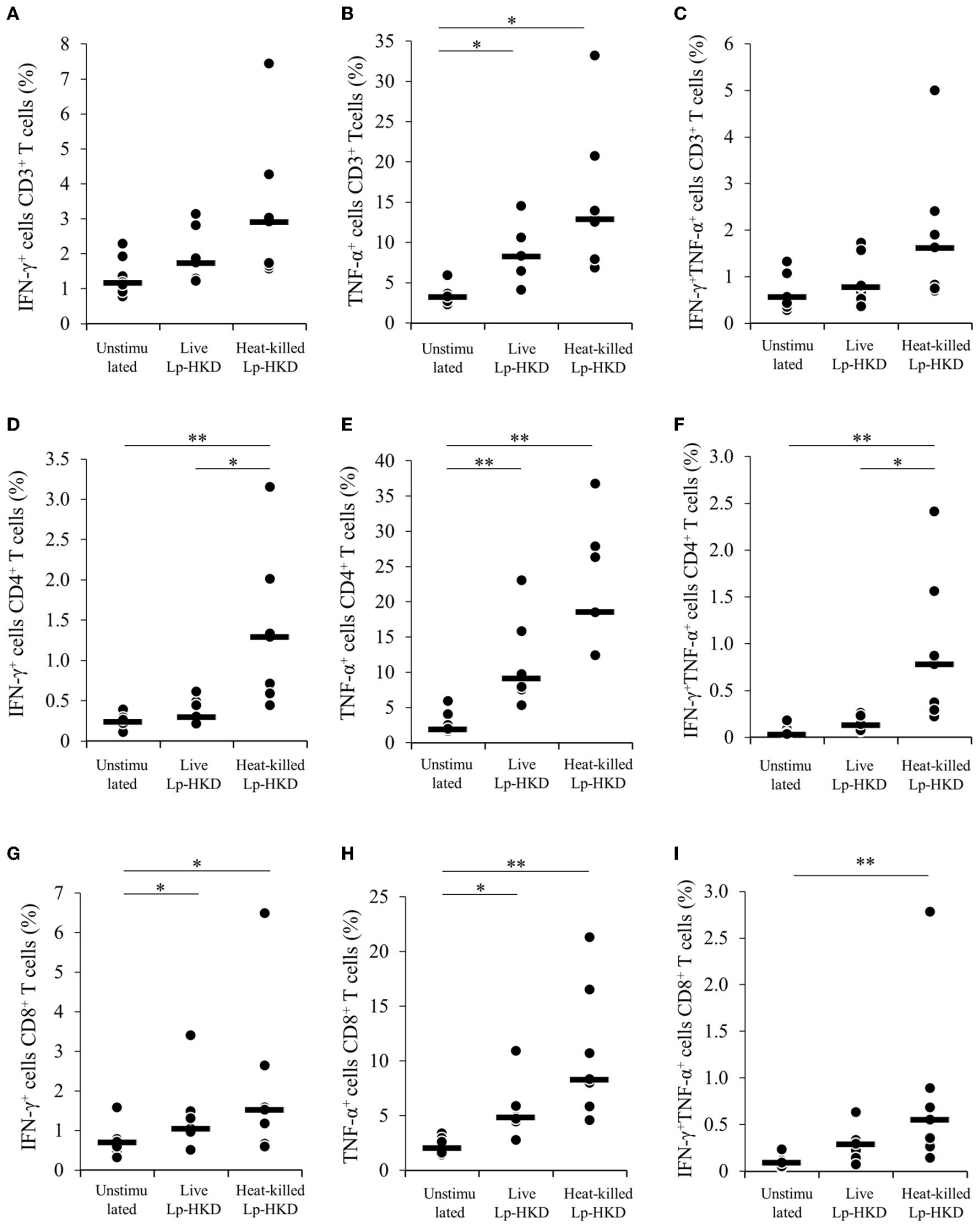
Upregulation of Th1 cytokine production in T cells by Lp-HKD. **(A–I)** PBMCs were incubated with live or heat-killed Lp-HKD (4 × 10^6^ CFU/mL) or without stimulation for 17 h. The expression of IFN-γ and TNF-α in CD3^+^ T cells [**(A–C)**, *n* = 7], CD3^+^CD4^+^ cells [**(D–F)**, *n* = 7], and CD3^+^CD8^+^ T cells [**(G–I)**, *n* = 7] was assayed by flow cytometry. Statistical significance was determined by the Steel-Dwass test. *p < 0.05, **p < 0.01.

To confirm whether Lp-HKD stimulation enhance Th1 responses, PBMCs were cultured either with live or heat-killed Lp-HKD, and the expression levels of Th1-related cytokines IFN-γ and TNF-α were analyzed in T cells by flow cytometry. As shown in Supplementary Figure S2, CD3^+^, CD3^+^CD4^+^, CD3^+^CD8^+^ T cells were gated in live lymphocytes and then analyzed for the expression of IFN-γ and TNF-α. TNF-α expression in CD3^+^ T cells was upregulated by treatment with live or heat-killed Lp-HKD (Figure 6B). The percentages of IFN-γ^+^ and IFN-γ^+^ TNF-α^+^ cells in CD3^+^ T cells were tended to be higher in PBMCs stimulated with live or heat-killed Lp-HKD than without Lp-HKD, although no significant differences were found (Figures 6A, C). Additionally, the percentages of IFN-γ^+^, TNF-α^+^, and IFN-γ^+^TNF-α^+^ cells in CD4^+^ T cells were increased when cultured with heat-killed Lp-HKD (Figures 6D–F). The expression of IFN-γ and TNF-α in CD8^+^ T cells was upregulated in PBMCs stimulated with live or heat-killed Lp-HKD (Figures 6G, H). Furthermore, the percentage of IFN-γ^+^TNF-α^+^CD8^+^ T cells was increased by the treatment with heat-killed Lp-HKD (Figure 6I). Taken together, Lp-HKD activates T cells and induces the production of Th1-related cytokines.

### Induction of antiviral effects by Lp-HKD

**Figure 7:**
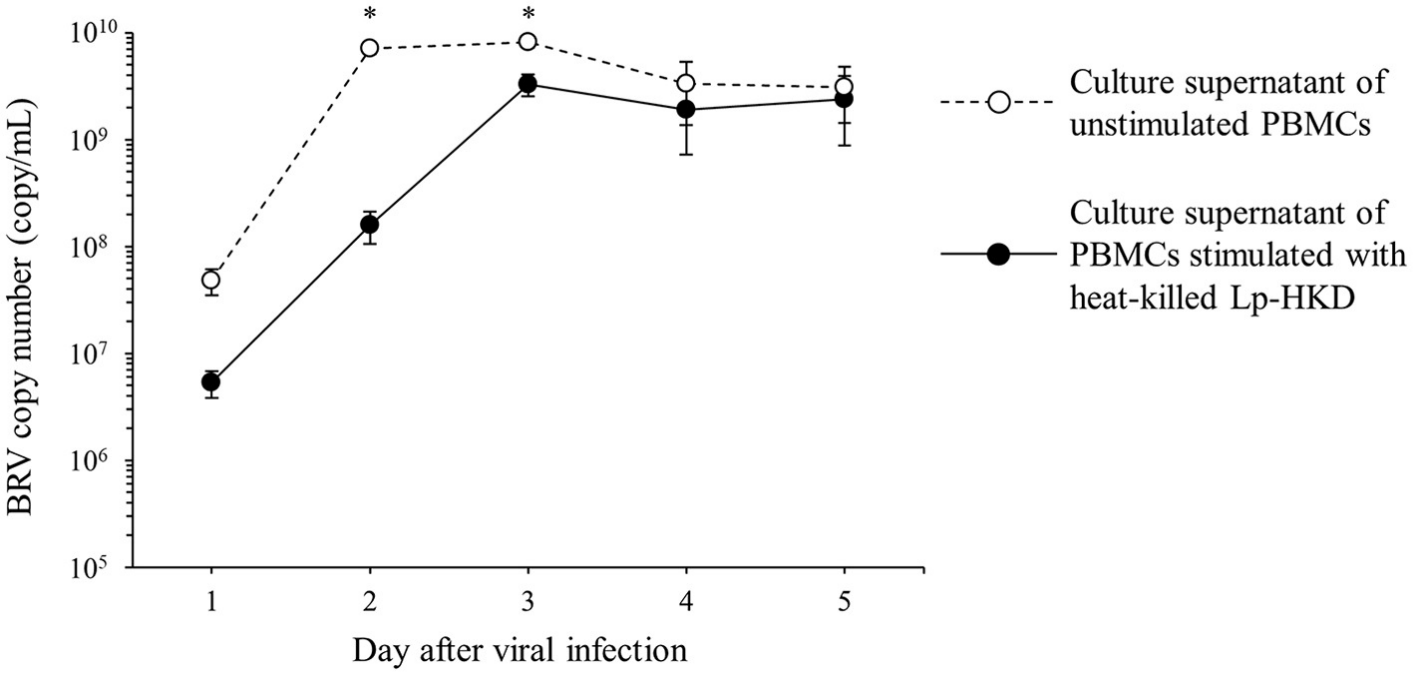
Induction of antiviral effects by Lp-HKD. MA104 cells were exposed to BRV and cultured in the presence of culture supernatants of PBMCs stimulated with or without heat-killed Lp-HKD (4 × 10^6^ CFU/mL). vRNA in the culture supernatants was quantitated by one-step qRT-PCR. Four independent wells were incubated for each sample, and the results were presented as the average of BRV copy numbers per 1 mL of culture supernatant. Statistical significance was determined by Welch's *t*-test. *p < 0.05.

To investigate whether the cytokines induced by Lp-HKD stimulation have antiviral effects against BRV, PBMCs were cultured with or without heat-killed Lp-HKD and the culture supernatants were added to BRV-infected MA104 cells. The culture supernatants of PBMCs stimulated with heat-killed Lp-HKD inhibited the proliferation of BRV at 2 and 3 days after BRV infection when compared to that of unstimulated PBMCs (Figure 7). These data suggest that cytokines produced by Lp-HKD-stimulated PBMCs had antiviral effects against BRV.

## Discussion

Calf diarrhea is a serious problem that increases economic losses in the dairy industry as a result of weight loss in surviving calves and sometimes calf mortality worldwide. Several probiotics have been used as preventive or supportive therapy for dairy cattle and neonatal calves for many years, and previous reports have indicated that they reduce the incidence and clinical signs of calf diarrhea (12, 20–30). A previous report indicated that Lp-HKD, a probiotic whose clinical study is ongoing in humans, decreased the intestinal pathology and the clinical sign of diarrhea in BRV-challenged calves (13). However, the mechanism by which Lp-HKD prevents severe diarrhea in BRV-infected calves remains to be elucidated. In humans, previous researches have demonstrated that probiotics treatment is effective against diarrhea caused by rotavirus in human. The rationale for using probiotics in acute infectious diarrhea is based on the assumption that they act against intestinal pathogens. Possible mechanisms of their effects include the stimulation of nonspecific and specific immune responses to pathogens (31, 32). Therefore, we hypothesized that Lp-HKD modulated bovine immune responses to viral infections including BRV infection in cattle. In this study, we revealed that Lp-HKD induced the production of IL-1β, IL-6, and IL-10 by CD14^+^ monocytes *via* the stimulation of TLR2 and TLR4. Since these cytokines have T-cell activating effects (33–36), it is suggested that cytokines produced by CD14^+^ monocytes stimulated by Lp-HKD could increase T-cell activation, leading to Th1 cytokine production.

Lipoteichoic acid (LTA) and peptidoglycan (PGN), which are components of the LAB cell wall, are recognized by TLR2, and the former is also recognized by TLR4 (37–39). Although the basic structure of cell walls is maintained in LAB, subtle structural differences between species and strains have been reported to result in different activations of TLR signaling pathways (40–42). A previous study on *L*. *plantarum*, K8, K88, K5-5, and K55-5 strains, which investigated the relationship between LTAs and their receptors, reported that the four strains of *L*. *plantarum* have different LTA structures, which contributed to different binding abilities with TLR2 and different immune activities in immune cells (43). Our current study demonstrated that the inhibition of TLR2 and TLR4 signaling by SsnB decreased the Lp-HKD-induced production of IL-1β and IL-6 but not of IL-10 (Figures 4A–F). Taken together, the results of this study suggest that Lp-HKD could predominantly induce IL-1β and IL-6 production by TLR2 and TLR4 signaling and IL-10 production by receptors other than TLR2 and TLR4. TLR9 recognizes unmethylated CpG DNA of bacteria (44, 45), and one of the TLR9 signaling pathways induces IL-10 production (46). Therefore, the induction of IL-10 production by Lp-HKD might be mediated by a TLR9 signaling pathway. Furthermore, in the current study, the TLR2/4 inhibition did not completely suppress the production of IL-1β and IL-6 (Figures 4A–D). These results indicate that Lp-HKD might be recognized by other receptors such as TLR9 which also promotes IL-1β and IL-6 expression (47). Further studies using extracts of cell wall components of Lp-HKD and the inhibitors of other receptors are warranted to confirm which signaling pathways in immune cells are activated by Lp-HKD.

The effect of immunomodulation by viable cells of probiotics is also obtained from the populations of dead cells (48). Taverniti and Guglielmetti (49) proposed the term “paraprobiotics” to indicate the use of inactivated microbial cells or cell fractions to confer health benefits. The emerging concern regarding safety problems arising from the use of live microorganisms in neonates and vulnerable populations is enhancing the interest in non-viable microorganisms (50, 51). For example, heat-killed *Lactobacillus acidophilus* LB strain is effective in the treatment of children with acute diarrhea and chronic diarrhea (52, 53). In the present study, the production of IL-1β, IL-6, and IFN-γ was significantly more induced by heat-killed Lp-HKD than by live Lp-HKD (Figures 1A, B, D). The microscopic examination of heat-killed Lp-HKD revealed no morphological change (data not shown). Abedi et al. (54) revealed that heat treatment altered the cell surface hydrophobicity of LAB and anticipated that the alternation influences the PGN structure in the cell wall. Therefore, structural changes of cell walls in Lp-HKD induced by heat treatment may lead to its better immune stimulating effect in heat-killed bacteria. Further research is required to evaluate the potential of the treatment of heat-killed Lp-HKD as paraprobiotics for calf diarrhea caused by BRV infection.

Previous studies have demonstrated that the regulation of antiviral cytokine pathways by probiotic administration is an important mechanism for the regulation of rotavirus (55). In this study, the Lp-HKD-induced production of antiviral cytokines suppressed the proliferation of BRV *in vitro* (Figure 7). Several reports demonstrated that IL-1 and IFN-γ inhibited the entry and replication of rotavirus *in vitro* (56, 57) and TNF-α induced an anti-rotavirus effect by the activation of classical nuclear factor-κB signaling (58). Hence, the present study suggests that the antiviral effect of Lp-HKD is presumably caused by the induction of IL-1β, IFN-γ, and TNF-α.

In this study, we evaluated the immune activating effects of Lp-HKD using PBMC cultivation assays *in vitro*. A previous paper reported the immune-stimulating effects of *Lactobacillus rhamnosus* GG strain and the correlation of the results of the immunological analyses *in vivo* and *in vitro* (59). Because we have not conducted the analysis of cytokine kinetics in blood and intestine after Lp-HKD treatment in BRV-challenged calves, further validation is needed to determine how immune responses are modulated in the intestinal tract of the Lp-HKD-treated calves. Large amounts of macrophages and dendritic cells were located at lamina propria in small intestine and have a potential to recognize the component of Lp-HKD *via* TLR pathways and induce the production of antiviral cytokines.

In conclusion, we found that Lp-HKD can induce the production of several cytokines, such as IL-1β, IL-6, and IL-10, by monocytes *via* TLR2 and TLR4, and these cytokines can activate T cells and the production of Th1 cytokines, such as IFN-γ and TNF-α. Thus, Lp-HKD has both immunostimulatory and antiviral effects, and Lp-HKD is expected to be applied as a preventive and therapeutic method against various infectious diseases including calf diarrhea.

## Data availability statement

The raw data supporting the conclusions of this article will be made available by the authors, without undue reservation.

## Ethics statement

The animal study was reviewed and approved by the Institutional Animal Care and Use Committee of Hokkaido University. Written informed consent was obtained from the owners for the participation of their animals in this study.

## Author contributions

SK, TO, NM, SM, and KO designed and supervised the project. MI and TO performed the experiments. MI, SK, TO, and NM analyzed the data and prepared the manuscript. KA, MH, TK, and YS contributed reagents, materials, or analysis tools. All authors reviewed and approved the manuscript.
