# Supplementary material for: In vitro evaluation of Lactiplantibacillus plantarum HOKKAIDO strain, effective lactic acid bacteria for calf diarrhea
Source: Front Vet Sci. 2023 Apr 5;10:1145445. doi: 10.3389/fvets.2023.1145445 (PMC10113454; doi:10.3389/fvets.2023.1145445)
Supplement: Supplementary file 1 [file Presentation_1.PPTX]

## Slide 1
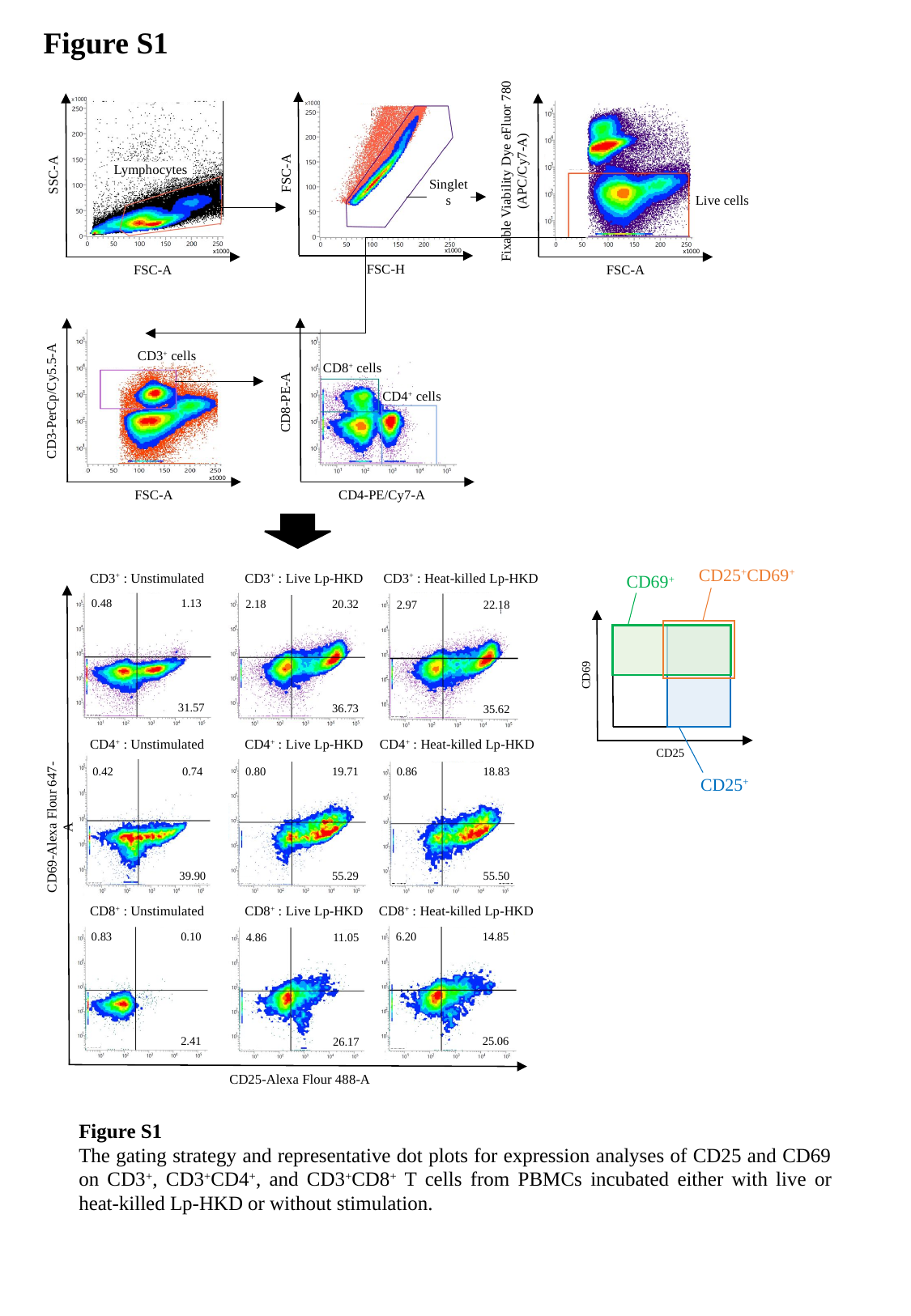

Figure S1
SSC-A
FSC-A
Fixable Viability Dye eFluor 780
(APC/Cy7-A)
FSC-A
Lymphocytes
Singlets
Live cells
x1000
x1000
x1000
FSC-H
FSC-A
CD8-PE-A
CD4-PE/Cy7-A
CD3-PerCp/Cy5.5-A
FSC-A
CD8+ cells
CD4+ cells
x1000
CD3+ cells
CD25+CD69+
CD69+
CD69
CD25
CD25+
CD3+ : Unstimulated
CD3+ : Live Lp-HKD
CD3+ : Heat-killed Lp-HKD
0.48
1.13
2.18
20.32
36.73
2.97
22.18
35.62
31.57
CD4+ : Unstimulated
CD4+ : Live Lp-HKD
CD4+ : Heat-killed Lp-HKD
0.42
0.74
39.90
0.86
18.83
55.50
0.80
19.71
55.29
CD69-Alexa Flour 647-A
CD8+ : Unstimulated
CD8+ : Live Lp-HKD
CD8+ : Heat-killed Lp-HKD
0.83
0.10
2.41
6.20
14.85
25.06
4.86
11.05
26.17
CD25-Alexa Flour 488-A
Figure S1
The gating strategy and representative dot plots for expression analyses of CD25 and CD69 on CD3+, CD3+CD4+, and CD3+CD8+ T cells from PBMCs incubated either with live or heat-killed Lp-HKD or without stimulation.

## Slide 2
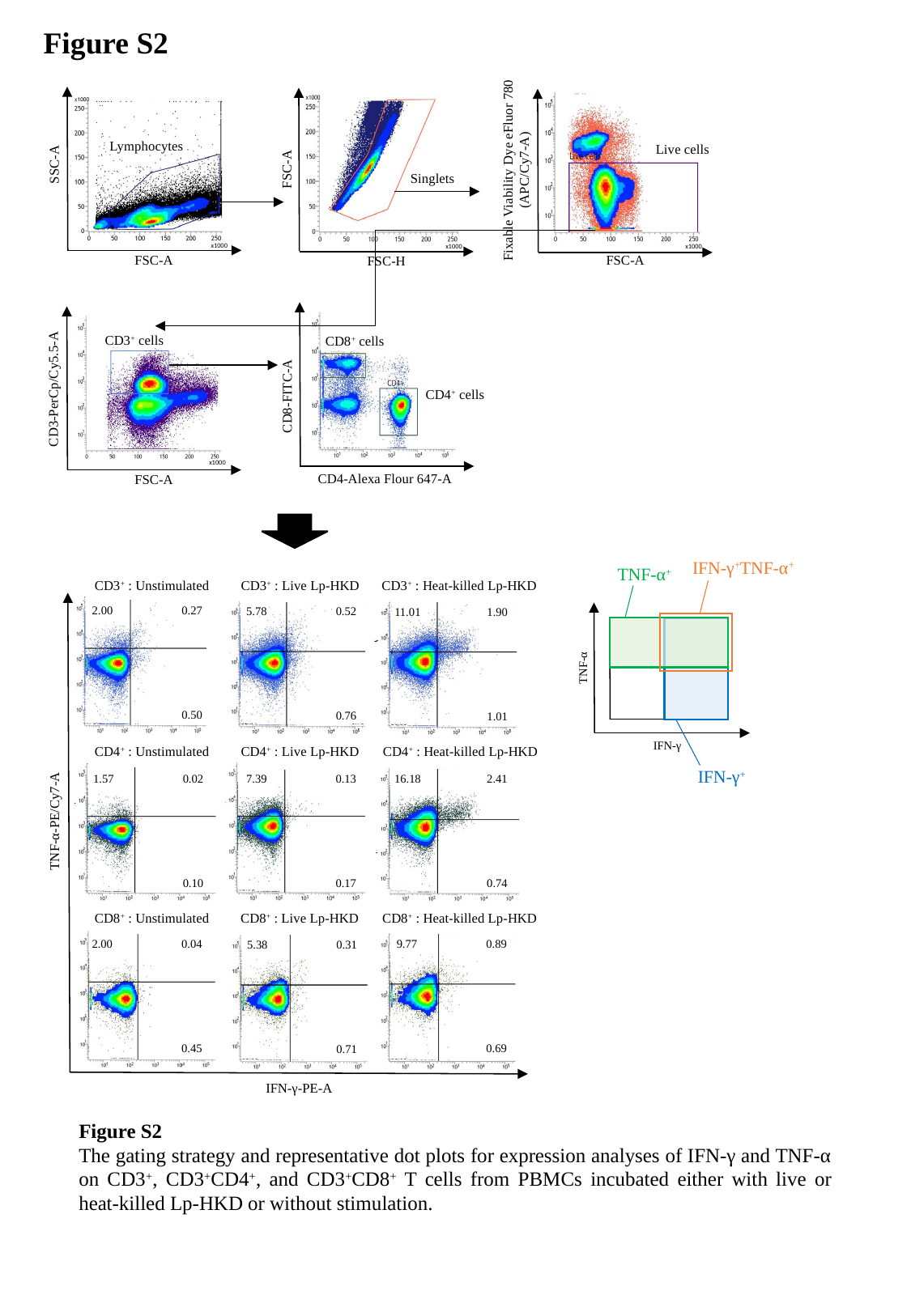

Figure S2
SSC-A
FSC-A
Lymphocytes
Live cells
Fixable Viability Dye eFluor 780
(APC/Cy7-A)
FSC-A
Singlets
x1000
x1000
x1000
FSC-A
FSC-H
CD8-FITC-A
CD4-Alexa Flour 647-A
CD3-PerCp/Cy5.5-A
FSC-A
CD3+ cells
CD8+ cells
CD4+ cells
x1000
IFN-γ+TNF-α+
TNF-α+
TNF-α
IFN-γ
IFN-γ+
CD3+ : Unstimulated
CD3+ : Live Lp-HKD
CD3+ : Heat-killed Lp-HKD
2.00
0.27
5.78
0.52
0.76
11.01
1.90
1.01
0.50
CD4+ : Unstimulated
CD4+ : Live Lp-HKD
CD4+ : Heat-killed Lp-HKD
1.57
0.02
0.10
16.18
2.41
0.74
7.39
0.13
0.17
TNF-α-PE/Cy7-A
CD8+ : Unstimulated
CD8+ : Live Lp-HKD
CD8+ : Heat-killed Lp-HKD
2.00
0.04
0.45
9.77
0.89
0.69
5.38
0.31
0.71
IFN-γ-PE-A
Figure S2
The gating strategy and representative dot plots for expression analyses of IFN-γ and TNF-α on CD3+, CD3+CD4+, and CD3+CD8+ T cells from PBMCs incubated either with live or heat-killed Lp-HKD or without stimulation.
